# Dementia Research in the Caribbean Hispanic Islands: Present Findings and Future Trends

**DOI:** 10.3389/fpubh.2020.611998

**Published:** 2021-01-18

**Authors:** Daisy Acosta, Jorge J. Llibre-Guerra, Ivonne Z. Jiménez-Velázquez, Juan J. Llibre-Rodríguez

**Affiliations:** ^1^Department of Internal Medicine, Universidad Nacional Pedro Henriquez Urena, Santo Domingo, Dominican Republic; ^2^Department of Neurology, Washington University School of Medicine in St. Louis, St. Louis, MO, United States; ^3^National Institute of Neurology and Neurosurgery, Habana, Cuba; ^4^Department of Internal Medicine, Medical Sciences Campus, University of Puerto Rico, San Juan, Puerto Rico; ^5^Finlay-Albarrán Medicine Faculty, Universidad de Ciencias Medicas, Habana, Cuba

**Keywords:** dementia, prevalence, incidence, Caribbean hispanics, regional policies

## Abstract

During the last decade, the Caribbean Hispanic islands experienced accelerated demographic aging, representing the fastest aging region within Latin America. Age-related non-communicable diseases, including dementia, are now reported at high prevalence. The Caribbean islands share similar genetic ancestry, culture, migration patterns, and risk profiles, providing a unique setting to understand dementia in the Caribbean-Hispanics. This perspective article aimed to describe the impact of dementia in the Caribbean, at a local and regional level and reflect on research strategies to address dementia. We report on 10/66 project findings, described research projects and regional plans for the region. According to our results, the prevalence of dementia in the Caribbean is the highest in Latin America, with 11.7% in Dominican Republic, 11.6% in Puerto Rico, and 10.8% in Cuba. Preliminary data from new waves of the 10/66 study shows increasing numbers of dementia cases. Furthermore, dementia is expected to be one of the most serious medical and social issues confronted by Caribbean health systems. However, there is a scarcity of knowledge, awareness, and health services to deal with this public health crisis. In light of the new evidence, local and regional strategies are underway to better understand dementia trends for the region and develop policies aimed to decrease the impact of dementia. Implementation of our national plans is critical to deal with an aging population with high dementia rates. Current recommendations include emphasizing public health prevention campaigns to address modifiable risk factors and expand support to caregiver and family interventions.

## Introduction

Epidemiological studies show a rapid increase in dementia in Hispanic populations (1, 2). Approximately twelve percent of older adults in the Latino population are diagnosed with Alzheimer's Dementia (AD), representing the highest growing proportion of AD cases in any ethnic group (3, 4). However, there is little to no understanding of disease onset, progression, and biomarker trajectories in Latino populations (5). Furthermore, most of the AD studies tend to include Latinos as a unique group, failing to sufficiently account for the real richness of linguistic, ethnic, ancestry, cultural, and socioeconomic diversity represented across Latino communities.

In relation to other Latino populations, Caribbean-Hispanics have several differences in customs, traditions, nutritional patterns, risk behaviors, and genetic admixtures, which may differentially impact the prevalence of dementia and expression of its symptoms. Furthermore, during the last decade, the Hispanic Caribbean islands (Cuba, Dominican Republic, and Puerto Rico) experienced accelerated demographic aging, representing the fastest aging region within Latin America, which posed a unique challenge for aging and dementia (6, 7). The Caribbean Hispanics represent 57.6% of the Caribbean population (Cuba = 11,326,616, Dominican Republic = 10,847,910, Puerto Rico = 3,193,694) region (6).

This perspective article aimed to examine the associations of genetics and socioeconomic determinants with dementia and describe the impact of dementia in the Hispanic Caribbean islands at a local and regional level. In addition, we present current research and describe future projects in the region. Finally, we share current dementia strategies aimed to address this epidemic within the Caribbean area.

## Aging and Dementia in the Caribbean Hispanic

The Caribbean is undergoing increasingly rapid population aging with the proportion of older persons (60 and over) increasing from 10% in 2000 to 14% in 2015, and projected to reach 25% by 2050 (6). The rate of demographic aging is substantially faster than occurred in western industrialized societies in the past (8, 9). Consequently, compared to High-Income Countries, the Caribbean region must adapt more quickly to aging populations, posing a challenge to more fragile economies and less prepared health care systems. Low fertility rates, a decrease in birth rate, and increased life expectancy due to medical advancements are the main factors related to the Caribbean's aging phenomenon (7). In addition, migration has played a unique role in the aging process within the Caribbean Hispanic region; the three islands have experienced extensive diasporas in the last century, with these large migrations—primarily to the United States (US)—establishing such well-known communities as Little Havana in Miami, Hispanic Harlem, and Washington Heights, in New York (10, 11). Recently, migration from PR to US increased after Hurricanes Irma and Maria (Sept 2017). Migrants are often young, and their departure has re-shape the population structure, as a result the age structure of the population have change significantly in the Caribbean islands.

As number and proportion of older persons increase, a central question to the Caribbean region is whether the aging of this population will be accompanied by sustained or improved health, better quality of life, and sufficient social and economic resources. To date, the increase in life expectancy has not been coupled with improvements in lifestyle; risk behaviors such as consuming high fat and carbohydrate diets, smoking, and sedentarism are becoming more common. Likewise, population aging has been associated with a sustained increase in non-communicable diseases, including neurodegenerative disorders, like dementia (2, 12). Dementia has become one of the most serious medical and social issues confronted by Caribbean health systems, with a markedly elevated prevalence and incidence compared to other Latin American countries in general (2).

**Figure 1:**
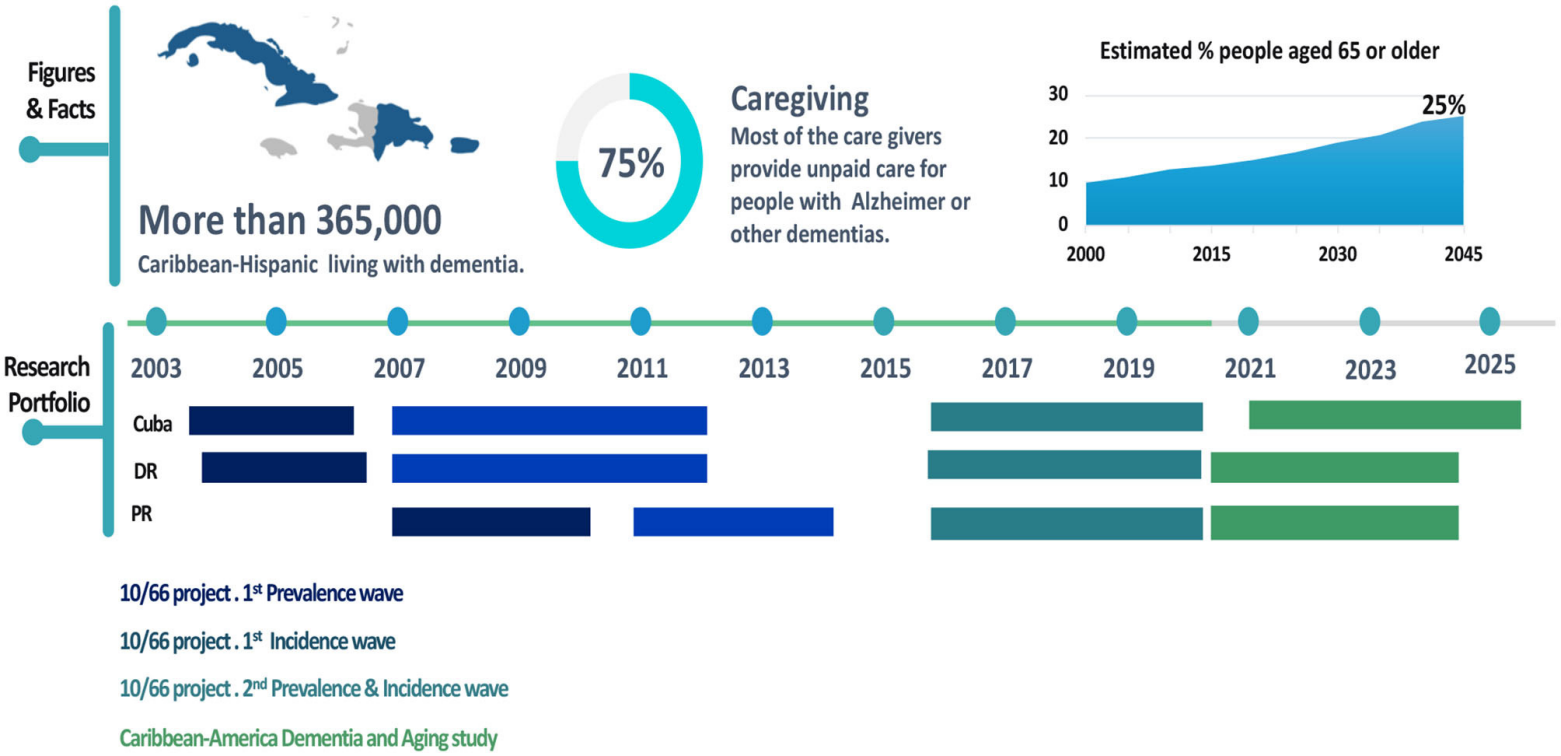
Dementia in the Caribbean Hispanic Islands: Research Agenda, Figures, and Fact.

Since 2003, the three islands in a coordinated effort led a major epidemiological study (Figure 1) to determine dementia prevalence, incidence, and impact across Caribbean countries using a validated and standard methodology (13). The studies were done under the umbrella of the 10/66 protocols (13), a multinational research initiative aimed to provide a detailed evidence to inform the development and implementation of policies for improving the health and social welfare of older people in low and middle income countries (14). The 10/66 studies encompass new methods to dementia research in Low and Middle Income countries (LMIC) by developing a novel approach to diagnosing dementia (the 10/66 Dementia Diagnosis) and addressing difficulties in making diagnoses among older people with little or no education and the use of standardized protocols across all sites (13, 15). Although, the 10/66 study included several Latin America countries, India, and China, in this perspective article we focus on the Hispanic Caribbean studies. Further details of the 10/66 studies have been described elsewhere (4, 16).

In the Caribbean region, the 10/66 project surveyed 6833 residents, aged 65 and over, in Cuba (*n* = 2813) and the Dominican Republic (*n* = 2011) from 2003 to 2007, and in Puerto Rico (*n* = 2009) from 2006 to 2008. The interviews were done in a single phase, and the methodology allowed for the diagnosis of dementia and its subtypes, as well as other mental disorders. Data obtained included sociodemographic characteristics, physical health, anthropometric measures; information about risk factors, disability, frailty, utilization of health services, income, lifestyle (including nutrition and exercise), care characteristics, caregiver burden, and genetic risk markers (including ApoE-4); implemented together with a physical and neurological examination. An interview with a reliable informant was required. An incidence phase (second wave) was conducted 3 to 4 years after cohort enrollment, 2007 to 2011. A third wave of the 10/66 protocol is currently underway (details of this new wave are provided below).

The 10/66 studies in the Caribbean (Figure 1) have led to several publications on the prevalence and incidence of dementia and other chronic diseases (hypertension, stroke, anemia, diabetes), the impact of dementia in terms of disability, dependency, economic cost, care arrangements, and access to services (17–20). We have focused our attention on determinants of longitudinal outcomes, specifically incident dementia, mortality, dependence, risk factors, and course of dementia/ and Mild Cognitive Impairment. According to our epidemiological studies, using the same methodology in all three countries, the prevalence of dementia in individuals 65 years old and over is ~11.7% in DR, 11.6% in the PR, and 10.8% in Cuba (Table 1). Preliminary data from a third wave of the 10/66 study suggest and in dementia prevalence relative to previous waves, probably related to a higher frequency of vascular risk factors and poor control of non-communicable diseases, including hypertension, diabetes, etc. With this continuing trend, by 2050, dementia in our countries may have one of the world's highest prevalence (with an estimated increase of 215%) (21, 22).

**Table 1:** Caribbean Hispanics Islands population characteristics and 10/66 cohort profile.

	**Cuba**	**Dominican Republic**	**Puerto Rico**
Population Size, (No) millions	11.1	10.6	3.2
Over age 65, (%)	15	7	15
Life expectancy at birth (years)	80	74	80
Fertility rate (birth per woman)	1.7	2.4	1.1
10/66 baseline characteristics			
Participants, No	2813	2011	2009
Response Rate (%)	94	95	93
Age (years), No (%)			
65-69	715 (25.5)	533 (26.5)	414 (20.6)
70-74	747(26.6)	520 (25.9)	456 (22.7)
75-79	618 (22.0)	396 (19.7)	483 (24.0)
>79	726 (25.9)	561 (27.9)	656 (12.0)
Gender (F), No (%)	1836 (65.3)	1325 (66)	1347 (67.3)
Dementia, No (%)	292 (10.4)	235 (11.7)	233 (11.6)
Mild Cognitive Impairment, No (%)	42 (1.5)	26 (1.3)	68 (3.4)
Stroke, No (%)	216 (7.7)	175 (8.7)	168 (8.4)
Hypertension, No (%)	1624 (57.9)	968 (48.6)	518 (32.1)

In the region, the main risk factors for dementia included: older age, family history of dementia, and lower levels of education. Poor cardiovascular health was associated with a higher incidence rate (23). Dementia is also among the top ten causes of death (4th-Puerto Rico, 6th-Cuba, 8th-Dominican Republic) (23, 24). However, due to the relative lack of early diagnosis and under registry on death certificates, current reports are likely to be under estimated.

Finally, the total direct and indirect costs of dementia was estimated to be 3.5 billion dollars, which is expected to double in 20 years (22).

As mentioned earlier, a third wave of assessments using an abbreviated form of the primary 10/66 survey is currently underway, ~10 years after the original baseline surveys (2016–2020). For the new survey (third wave, *n* = 6000), we are revisiting the original catchment areas to generate a revised representative estimate of the prevalence of dementia for the residents who are 65 years old or older; the updated sample includes those individuals who, since the first wave of the survey have been incorporated into the age group of interest, either from having aged into it or from having moved into the areas. The new, third-wave prevalence survey includes a comprehensive assessment of health status, which will consist of spirometry, body mass index, visual acuity, grip strength, and tests to determine the presence of hearing impairment (16). The new wave aim to determine the changes in prevalence and incidence of dementia in the region and its associated risk factors—emphasizing cardiovascular issues—and to evaluate probable associations with APOE gene, markers of inflamation and immunusenescence. Moreover, a nested cohort of 300 individuals was randomly selected (150 with a high risk of incident dependence and 150 without) aimed to explore markers of frailty, including extensive laboratory testing of frailty biomarkers. This sub-group will be followed 18 months later to re-assess vital status, needs for care, disability, cognitive function, and significant health-related life events in the intervening period.

## Population Admixture, Genetics, and Caribbean Hispanics

The Caribbean Hispanic population is highly multiethnic, reflecting its complex colonial origins. Intermarriage between diverse groups is widespread, and estimates of the percentage of African descent people vary enormously, ranging from 34 to 62% (population genetic admixture includes mainly European and African ancestry, with little contribution of Indigenous ancestry) (25, 26). This genetic ancestry pattern is different from the one described in South America, which includes predominantly European and Indigenous populations (27). The differences in genetics between the Caribbean-Hispanic and non-Caribbean Hispanics may yield relevant information regarding the influence of admixture background on dementia risk. For example, the risk of AD associated with the *APOE-*ε*4* allele has been found to vary according with African ancestry proportions (28, 29).

According to our studies, the association between dementia and *APOE-*ϵ*4* is weaker in Caribbean Hispanics compared to Caucasian populations. In the three Caribbean islands being an *APOE-*ϵ*4* carrier increases the risk to develop dementia by two folds (SHR 2.03 [1.32-3.11]); in Caucasian populations the risk among *APOE-*ϵ*4* carriers is 10 to 15 times higher than in non *APOE-*ϵ*4* carriers (28, 30, 31). To date only 3% of genetics studies in AD have been done in Hispanics population; (32, 33) like *APOE-*ϵ*4*, other genetic variants previously described in Caucasians (e.g., *TREM2, SORL1, ABCA7*) may have a differential risk among the Hispanic population; therefore the influences of genetic risk factors in Caribbean Hispanics should be explored in future studies.

The frequency of Dominantly Inherited Alzheimer disease (DIAD) is relatively high in the Caribbean countries compare to Europe and US; (33, 34) which can be attributable to common ancestors causing a founder effect during early colonization periods (35, 36). It is very likely that the establishment of new colonies in the Caribbean islands combined with a high degree of inbreeding among Caribbean populations (37) created a reduced amount of genetic variation within the new population settlements, influencing the spread of these mutations across the islands. In Cuba, Bertoli et al. (38) described a novel Presenilin 1 (PSEN1) pathogenic variant (*PSEN1_L174M*) affecting several families (280 family members) in the Cuban western provinces. Furthermore, two decades ago, a family study was developed in New York that included multiplex relatives affected with AD in Dominican Republic and Puerto Rico, leading to the discovery of novel *PSEN1* pathogenic variant *(PSEN1_G206A)* (36). Since 2008, a new Early Onset AD study has been conducted in Puerto Rico, leading to the discovery of 91 unrelated families featuring this pathogenic variant. A total of 682 family members have been evaluated and followed in one of the world's biggest cohorts of early-onset families (37, 39). Several studies, observational and clinical trials with novel medications are ongoing in Puerto Rico for family members of the PSEN1 families, as well as for sporadic AD cases.

## Regional and Local Policies in the Caribbean

### Education and Training in Dementia

As mentioned early, the prevalence of dementia is high among use Caribbean-Hispanics, yet knowledge, health services, and awareness of the disease are scarce (40). Awareness and early diagnosis are crucial elements to reduce dementia's impact, benefit patients and caregivers, and reduce treatment costs (41). Early detection can prompt evaluation for reversible causes, improve the care of comorbid illnesses, guide the selection of appropriate symptomatic therapies, and identify the needs for social support (41–43).

Unfortunately, cognitive impairment and dementia diagnosis depend mostly on clinical suspicion, and ~50% of dementia cases are missed in primary care, delaying detection until later in the disease course. Furthermore, awareness about dementia diagnosis and care is not only relevant for health care professionals, but also for the general population, especially among those caring for someone with dementia; and these are unmet needs in LMIC (44, 45). Consequently, there is a current need to increase healthcare providers and caregivers' training options.

Several local and regional strategies have been implemented to address these issues. The Alzheimer's Research Center in Cuba has developed a multidisciplinary Master's degree program to improve early diagnosis, treatment, and support of people living with dementia (46). Through this program, trainees will gain the necessary knowledge to advance research on the dementia field and provide better care to dementia patients and their families. Similar strategies are underway in the Dominican Republic and Puerto Rico. For example, the Department of mental health of the Ministry of health in the Dominican Republic for the last 4 years has been training the primary care workforce in the mhGAP intervention guide (47) with the aim that by 2025, at least 50% of the estimated number of people with dementia will receive an accurate diagnosis.

Another need is to expand the training of caregivers. The regional Alzheimer Associations have led a caregiver awareness program to improve caretaking abilities and expand caregivers' support. A full training video-series featuring relevant topics regarding caretaking in dementia and advice from health care professionals are available to view and share free of charge. “Conversando con Los Cuidadores”—new series in Spanish- includes eight videos focusing on critical issues related to the work and role of those who provide care for relatives with dementia. Training of caregivers will enhance their management ability and enables them to handle disruptive situations. The Dominican Alzheimer's Association has been developing online and on-site training sessions to support and decrease the burden associated with caretaking. The 10/66 “Helping Caregivers to Care, train-the-trainer” intervention was tested in a randomized control trial in the DR, providing evidence on the relevance of caregiver training to decrease the burden associated with care. This intervention continues to be widely used by the Dominican Alzheimer's Association to train caregivers (alz.co.uk/helping-carers-to-care). The University of Puerto Rico, Geriatric's Education Center, offers specialized courses to train caregivers in different sites, including a special arrangement with the Dominican Republic Embassy in PR, to train Dominican caregivers working in Puerto Rico. Likewise, there are multiple caregiver training programs in private universities on the island.

## Local and Regional Plans to Address Dementia

In 2015, Cuba and the Alzheimer's Research Center announced the National plan for dementia, and it was adopted as a National dementia strategy by the Cuban Ministry of health in 2016 (23). The main goals of the Cuban national strategy included: 1—Increase awareness, information, education, and support for families; 2—Risk reduction (e.g., better control of cardiovascular risk factors, reduce physical inactivity, promote healthy diets among others); 3—Timely diagnosis and access to treatment; 4—Improve care for dementia patients and their families; 5—Reduce stigma around dementia; 6—Increase professional development and train families for patient care; 7—Promote basic, clinical and epidemiological research on dementia; 8—Familiarize health teams with laws protecting the rights of older adults and people with cognitive impairment; 9—Assess and improve the quality of health care, social care, and long-term care support and services. Furthermore, Cuba has taken the first steps in implementing the Global action plan on the public health response to dementia 2017–2025.

Similar to Cuba, in 2015, Puerto Rico launched the *Alzheimer's disease Action Plan* in response to the World Health Organization's (WHO) call to action, by rating AD and other dementias as a public health priority. The focal areas of the PLAN were divided into seven pillars: (1) Public Policy (2) Public Health Efforts and Epidemiologic Surveillance (3) Home and Community Caregiving Services (4) Education and Training (5) Diagnosis and Treatment, (6) Long Term Care Services, and (7) Long-Term Care Financing. Each area includes specifics goals to be accomplished by 2025.

Finally, in July 2020, the Dominican Republic's Ministry of Public Health, in close partnership with Asociación Dominicana de Alzheimer, announced a national strategy for dementia with similar action points as Cuba and Puerto Rico. Future efforts in the region are needed to achieve a coordinated response and plan to address the rising number of dementia cases.

## Upcoming Research Efforts

The Caribbean American Dementia and Aging Study (CADAS) is new research study funded through the National Institute of Health (R01AG064778) aimed to support regional research efforts to expand the previous waves of 10/66 studies to rural populations and facilitate a nationwide dementia estimate (Figure 1). Results from this project will be further compared with databases of Hispanic data collected in mainland USA, including the Health and Retirement Study (HRS) and Harmonized Cognitive Assessment Protocol (HCAP). In addition, in collaboration with the Alzheimer's Association, several countries in the region will launch the LatAm FINGERS, a multi-domain lifestyle intervention (diet and exercise), aimed to improve cognitive function (48). This study will be conducted from 2020 to 2022 and will recruit 1,300 participants from 13 countries in Latin America, including Argentina, Brazil, Bolivia, Chile, Colombia, Costa Rica, Cuba, Dominican Republic, Ecuador, Mexico, Paraguay, Peru, Puerto Rico, and Uruguay; details of LatAm FINGERS has been described elsewhere (48). Both CADAS and LatAm fingers projects show the relevance of international collaboration to push regional and locals research agendas.

To better understand disease pathology and AD progression in Caribbean-Hispanics, a new Brain Bank is already in place in DR, another one is being organized in PR. Our final goal is to develop a Hispanic Caribbean Coordinating Center for neuropathology sample collection and storage. To maintain a coordinated research program, Caribbean researchers are meeting several times per year, promoting the work's continuity and keeping the international collaborations. More recently, the Ministry of Higher Education Science and Technology of the Dominican Republic awarded the Brain Bank research funding to expand research on AD pathology, with a special focus in Tau patho-physiology. This project is a joint consortium with a local university, “Pontificia Universidad Catolica Madre y Maestra” and Universidad Autonoma de Mexico (UNAM).

## Conclusion

Dementia represents a challenge for public health in developing countries with rapid demographic transitions, such as those occurring in the Caribbean. We propose expanding our research efforts to rural areas and explore gene by environment interactions on dementia risk. Emphasis on genetic family studies and admixture and new clinical trials with Hispanic representation, should continue. The development of brain banks and the implementation of biomarker studies are current research priorities. Implementation of our national plans is critical to deal with an aging population with high dementia rates, ideally a true public health-focused approach should aim at social change and emphasize on public-health prevention campaigns directed to addressing modifiable risk factors and developing caregiver and family interventions.

## Data Availability Statement

Publicly available datasets were analyzed in this study. This data can be found here: https://1066.alz.co.uk/.

## Ethics Statement

The studies involving human participants were reviewed and approved by Universidad Nacional Pedro Henriquez Ureña, Universidad de Ciencias Médicas de la Habana, University of Puerto Rico. The patients/participants provided their written informed consent to participate in this study.

## Author Contributions

All authors had full access to all the data in the study and take responsibility for the integrity of the data and the accuracy of the data analysis. JL-R, JL-G, IJ-V, and DA: study concept and design. JL-R, IJ-V, and DA: acquisition, analysis, or interpretation of data. JL-G and DA: drafting of the manuscript. All authors critical revision of the manuscript for important intellectual content. JL-G: project administration. JL-R: study supervision.

## Conflict of Interest

The authors declare that the research was conducted in the absence of any commercial or financial relationships that could be construed as a potential conflict of interest.

## References

[1] Nitrini R Bottino CMC Albala C Custodio Capuñay NS Ketzoian C Llibre Rodriguez JJ. Prevalence of dementia in Latin America: a collaborative study of population-based cohorts. Int Psychogeriatrics. (2009) 21:622–30. 10.1017/S1041610209009430 19505354 PMC8324310

[2] Rodriguez JJL Ferri CP Acosta D Guerra M Huang Y Jacob KS. Prevalence of dementia in Latin America, India, and China: a population-based cross-sectional survey. Lancet. (2008) 372:464–74. 10.1016/S0140-6736(08)61002-8 18657855 PMC2854470

[3] Rocca WA Petersen RC Knopman DS Hebert LE Evans DA Hall KS. Trends in the incidence and prevalence of Alzheimer's disease, dementia, and cognitive impairment in the United States. Alzheimers Dement. (2011) 7:80–93. 10.1016/j.jalz.2010.11.002 21255746 PMC3026476

[4] Prina AM Mayston R Wu Y-T Prince M A review of the 10/66 dementia research group. Soc Psychiatry Psychiatr Epidemiol. (2018) 2018:1-10. 10.1007/s00127-018-1626-7 PMC6336743 30467589

[5] González HM Tarraf W Schneiderman N Fornage M Vásquez PM Zeng D. Prevalence and correlates of mild cognitive impairment among diverse hispanics/Latinos: study of latinos-Investigation of neurocognitive aging results. Alzheimer's Dement. (2019) 15:1507–15. 10.1016/j.jalz.2019.08.202 31753701 PMC7318558

[6] Quashie NT Jones F Gény LR Abdulkadri A Population ageing and sustainable development in the Caribbean: where are we 15 years post MIPAA. Int J Ageing Dev Countries. (2018) 2:128–48.

[7] Caribbean SC for the W on S the SF for P to M the C of A in LA the Population C on Education D of B SS and The National Academies of Sciences E M Aging in Latin America and the Caribbean in Global Perspective. (2015).

[8] United Nations Department of Economic and Social Affairs Population Division World Population Ageing 2019: Highlights (ST/ESA/SER.A/430). (2019).

[9] Kinsella K Strengthening the Scientific Foundation for Policymaking to Meet the Challenges of Aging in Latin America and the Caribbean: Summary of a Workshop. Washington, DC: The National Academies Press (2015). 26512392

[10] Moya JC A continent of immigrants: postcolonial shifts in the Western Hemisphere. Hisp Am Hist Rev. (2006) 86:1–28. 10.1215/00182168-86-1-1

[11] Rumbaut RG The making of a people. In: M. Tienda, F. Mitchell, editors. Hispanics and the Future of America. National Academies Press (2006). p. 16–65. Available online at: https://ssrn.com/abstract=1877405. 20669436

[12] Molero AE Pino-Ramírez G Maestre GE. High prevalence of dementia in a caribbean population. Neuroepidemiology. (2007) 29:107–12. 10.1159/000109824 17940342

[13] Prince MJ De Rodriguez JL Noriega L Lopez A Acosta D Albanese E. The 10/66 dementia research group's fully operationalised DSMIV dementia computerized diagnostic algorithm, compared with the 10/66 dementia algorithm and a clinician diagnosis: a population validation study. BMC Public Health. (2008) 8:219. 10.1186/1471-2458-8-219 18577205 PMC2474864

[14] Prince MJ The 10/66 dementia research group - 10 years on. Indian J Psychiatry. (2009) 51(Suppl 1):S8–S15. PMC3038536 21416024

[15] Prince M Ferri CP Acosta D Albanese E Arizaga R Dewey M The protocols for the 10/66 dementia research group population-based research programme. BMC Public Health. (2007) 7:165 10.1186/1471-2458-7-165 17659078 PMC1965476

[16] Prina AM Acosta D Acostas I Guerra M Huang Y Jotheeswaran AT. Cohort profile: the 10/66 study. Int J Epidemiol. (2016) 46:dyw056. 10.1093/ije/dyw056 27154633 PMC5837706

[17] Sousa RM Ferri CP Acosta D Guerra M Huang Y Jacob K. The contribution of chronic diseases to the prevalence of dependence among older people in latin america, china and india: a 10/66 dementia research group population-based survey. BMC Geriatr. (2010) 10:53. 10.1186/1471-2318-10-53 20691064 PMC2923155

[18] Llibre JDJ López AM Valhuerdi A Guerra M Llibre-Guerra JJ Sánchez YY. Frailty, dependency and mortality predictors in a cohort of Cuban older adults, 2003-2011. MEDICC Rev. (2014) 16:24–30. 10.37757/MR2014.V16.N1.6 24487672

[19] Prince M Acosta D Ferri CP Guerra M Huang Y Rodriguez JJL. Dementia incidence and mortality in middle-income countries, and associations with indicators of cognitive reserve: a 10/66 dementia research group population-based cohort study. Lancet. (2012) 380:50–8. 10.1016/S0140-6736(12)60399-7 22626851 PMC3525981

[20] Pasquini L Llibre Guerra J Prince M Chua K-C Prina AM. Neurological signs as early determinants of dementia and predictors of mortality among older adults in Latin America: a 10/66 study using the NEUROEX assessment. BMC Neurol. (2018) 18:163. 10.1186/s12883-018-1167-4 30285663 PMC6168999

[21] Coste EL Prevalencia LA Alzheimer D Demencia DE Informe ADI/Bupa. La Demencia en América: el Coste y la Prevalencia del Alzheimer y Otros Tipos de Demencia. (2013).

[22] Prince M Wimo A Guerchet M Gemma-Claire A Wu Y-T. World Alzheimer Report 2015: The Global Impact of Dementia - An Analysis of Prevalence, Incidence, Cost and Trends. London: Alzheimer's Disease International (2015). p. 84. Available online at: http://www.worldalzreport2015.org/

[23] Llibre-Rodríguez J de J Valhuerdi-Cepero A López-Medina AM Noriega-Fernández L Porto-Álvarez R Guerra-HernóndezMA. Cuba's aging and Alzheimer longitudinal study. MEDICC Rev. (2017) 19:31–5. 10.37757/MR2017.V19.N1.6 28225543

[24] Figueroa R Steenland K MacNeil JR Levey AI Vega IE. Geographical differences in the occurrence of Alzheimer's disease mortality: United States versus puerto rico. Am J Alzheimers Dis Other Demen. (2008) 23:462–9. 10.1177/1533317508321909 18955725 PMC3640495

[25] Moreno-Estrada A Gravel S Zakharia F McCauley JL Byrnes JK Gignoux CR Reconstructing the population genetic history of the caribbean. PLoS Genet. (2013) 9:e1003925 10.1371/journal.pgen.1003925 24244192 PMC3828151

[26] Homburger JR Moreno-Estrada A Gignoux CR Nelson D Sanchez E Ortiz-Tello P. Genomic insights into the ancestry and demographic history of South America. PLoS Genet. (2015) 11:e1005602. 10.1371/journal.pgen.1005602 26636962 PMC4670080

[27] Mao X Bigham AW Mei R Gutierrez G Weiss KM Brutsaert TD. A genomewide admixture mapping panel for hispanic/Latino populations. Am J Hum Genet. (2007) 80:1171–8. 10.1086/518564 17503334 PMC1867104

[28] Teruel BM Rodríguez JJL McKeigue P Mesa T TC Fuentes E Cepero A AV. Interactions between genetic admixture, ethnic identity, APOE genotype and dementia prevalence in an admixed cuban sample; a cross-sectional population survey and nested case-control study. BMC Med Genet. (2011) 12:43. 10.1186/1471-2350-12-43 21435264 PMC3079615

[29] Rajabli F Feliciano BE Celis K Hamilton-Nelson KL Whitehead PL Adams LD. Ancestral origin of apoE ε4 Alzheimer disease risk in puerto rican and african american populations. PLoS Genet. (2018) 14:e1007791. 10.1371/journal.pgen.1007791 30517106 PMC6281216

[30] Blue EE Horimoto ARVR Mukherjee S Wijsman EM Thornton TA. Local ancestry at aPOE modifies alzheimer's disease risk in Caribbean hispanics. Alzheimers Dement. (2019) 15:1524–32. 10.1016/j.jalz.2019.07.016 31606368 PMC6925639

[31] Cruchaga C Del-Aguila JL Saef B Black K Fernandez MV Budde J. Polygenic risk score of sporadic late-onset Alzheimer's disease reveals a shared architecture with the familial and early-onset forms. Alzheimer's Dement. (2018) 14:205–14. 10.1016/j.jalz.2017.08.013 28943286 PMC5803427

[32] Kunkle BW Grenier-Boley B Sims R Bis JC Damotte V Naj AC Genetic meta-analysis of diagnosed Alzheimer's disease identifies new risk loci and implicates a, tau, immunity and lipid processing. Nat Genet. (2019) 51:414–30. 10.1038/s41588-019-0358-2 30820047 PMC6463297

[33] Llibre-Guerra JJ Li Y Allegri RF Mendez PC Surace EI Llibre-Rodriguez JJ Dominantly inherited Alzheimer's disease in Latin America: genetic heterogeneity and clinical phenotypes. Alzheimer's Dement. (2020) 2020:alz12227. 10.1002/alz.044794 PMC8140610 33226734

[34] Cruts M Van Duijn CM Backhovens H Van Den Broeck M Wehnert A Serneels S. Estimation of the genetic contribution of presenilin-1 and−2 mutations in a population-based study of presenile alzheimer disease. Hum Mol Genet. (1998) 7:43–51. 10.1093/hmg/7.1.43 9384602

[35] DeGiorgio M Jakobsson M Rosenberg NA. Explaining worldwide patterns of human genetic variation using a coalescent-based serial founder model of migration outward from Africa. Proc Natl Acad Sci USA. (2009) 106:16057–62. 10.1073/pnas.0903341106 19706453 PMC2752555

[36] Athan ES Williamson J Ciappa A Santana V Romas SN Lee JH. A founder mutation in presenilin 1 causing early-onset Alzheimer disease in unrelated Caribbean hispanic families. J Am Med Assoc. (2001) 286:2257–63. 10.1001/jama.286.18.2257 11710891

[37] Vardarajan BN Schaid DJ Reitz C Lantigua R Medrano M Jiménez-Velázquez IZ. Inbreeding among Caribbean hispanics from the dominican republic and its effects on risk of Alzheimer disease. Genet Med. (2015) 17:639–43. 10.1038/gim.2014.161 25394174 PMC4430451

[38] Bertoli Avella AM Marcheco Teruel B Llibre Rodriguez JJ Gomez Viera N Borrajero Martinez I Severijnen EA. A novel presenilin 1 mutation (L174 m) in a large cuban family with early onset Alzheimer disease. Neurogenetics. (2002) 4:97–104. 10.1007/s10048-002-0136-6 12484344

[39] Lee JH Cheng R Vardarajan B Lantigua R Reyes-Dumeyer D Ortmann W. Genetic modifiers of age at onset in carriers of the g206A mutation in pSEN1 with familial Alzheimer disease among Caribbean hispanics. JAMA Neurol. (2015) 72:1043–51. 10.1001/jamaneurol.2015.1424 26214276 PMC5010776

[40] Prince M Bryce R Albanese E Wimo A Ribeiro W Ferri CP. The global prevalence of dementia: a systematic review and metaanalysis. Alzheimers Dement. (2013) 9:63–75.e2. 10.1016/j.jalz.2012.11.007 23305823

[41] Langa KM Levine DA. The diagnosis and management of mild cognitive impairment: a clinical review. JAMA. (2014) 312:2551–61. 10.1001/jama.2014.13806 25514304 PMC4269302

[42] Robinson L Tang E Taylor JP. Dementia: timely diagnosis and early intervention. BMJ. (2015) 350:3029. 10.1136/bmj.h3029 26079686 PMC4468575

[43] Ashford JW Borson S O'Hara R Dash P Frank L Robert P Should older adults be screened for dementia? It is important to screen for evidence of dementia! *Alzheimer's Dement* (2007) 3:75–80. 10.1016/j.jalz.2007.03.005 PMC2804947 19595920

[44] Parra MA Baez S Allegri R Nitrini R Lopera F Slachevsky A. Dementia in Latin America assessing the present and envisioning the future. Neurology. (2018) 90:222–31. 10.1212/WNL.0000000000004897 29305437 PMC5791795

[45] Gonzalez FJ Gaona C Quintero M Chavez CA Selga J Maestre GE. Building capacity for dementia care in Latin America and the Caribbean. Dement Neuropsychol. (2014) 8:310–6. 10.1590/S1980-57642014DN84000002 25932285 PMC4412169

[46] Ms RIB Drsc JJLMPH Ms AF. Cuba ' s strategy for Alzheimer disease and dementia syndromes. MEDICC Rev. (2016) 2016:9-13. 10.37757/MR2016.V18.N4.2 27829648

[47] Keynejad RC Dua T Barbui C Thornicroft G. WHO mental health gap action programme (mhGAP) intervention guide: a systematic review of evidence from low and middleincome countries. Evid Based Ment Health. (2018) 21:29–33. 10.1136/eb-2017-102750 28903977 PMC10283403

[48] Kivipelto M Mangialasche F Snyder HM Allegri R Andrieu S Arai H. World-wide FINGERS network: a global approach to risk reduction and prevention of dementia. Alzheimer's Dement. (2020) 16:1078–94. 10.1002/alz.12123 32627328 PMC9527644

